# Characterization of the Immunogenomic Landscape of Ovarian Cancer Uncovers a Distinct Subset of Endometroid Tumors Associated with High *CST2* Expression and a Favorable Prognosis

**DOI:** 10.1158/2767-9764.CRC-25-0150

**Published:** 2026-01-28

**Authors:** James Boyle, Jan Zaucha, Felicia Ng, Andreas Spitzmüller, Miljenka Vuko, Felix J. Segerer, Monica Azqueta-Gavaldon, Marco Testori, Amelia Raymond, Barrett R.B. Nuttall, Andrea Ennio Storti, Sakshi Gulati, Shaan Gill, Michael Surace, Paola Marco-Casanova, Emma V. Jones, Ben S. Sidders, Jorge S. Reis-Filho, Maurizio Scaltriti, Anita Semertzidou, Helen K. Angell

**Affiliations:** 1 Oncology Data Science, Research and Early Development, Oncology R&D, AstraZeneca, Cambridge, United Kingdom.; 2 Computational Pathology GmbH, AstraZeneca, Munich, Germany.; 3 Translational Medicine, Oncology R&D, AstraZeneca, Boston, Massachusetts.; 4 Oncology Data Science Platforms, Oncology R&D, AstraZeneca, Munich, Germany.; 5 Oncology Data Science Platforms, Oncology R&D, AstraZeneca, Cambridge, United Kingdom.; 6 Translational Medicine, Oncology R&D, AstraZeneca, Cambridge, United Kingdom.; 7 Translational Medicine, Oncology R&D, AstraZeneca, Gaithersburg, Maryland.; 8 Cancer Biomarker Development, Oncology R&D, AstraZeneca, Gaithersburg, Maryland.

## Abstract

Ovarian cancers with similar histopathologic but diverse immunogenomic profiles may require different treatment regimens to achieve the best clinical outcomes. The development of optimal treatment regimens will thus require an understanding of the main ways in which ovarian cancers differ with respect to their immunogenomic profiles. We used a multimodal latent variable model to enable an integrated analysis of somatic mutation, mRNA expression, and multiplex immunofluorescence data to uncover the principal drivers of intertumor heterogeneity across 197 patients with ovarian cancer. We found that the majority of the immunogenomic intertumor heterogeneity was driven by gene expression and immune infiltration in the tumor core and invasive margin. Moreover, much of this heterogeneity could not be explained by histologic subtype; somatic mutation patterns explained much of the difference between high-grade serous and other subtypes. Clustering of samples according to their positions in the latent space revealed a distinct subgroup of endometrioid ovarian cancer tumors, characterized by increased CST2 expression and improved prognosis, among other immunogenomic features. We identified a group of collagen-related genes, for which expression in all subtypes was inversely correlated with the density of proliferating tumor cells in the tumor core and associated with increased levels of inflammatory fibroblasts, independent of tumor purity. In summary, our findings advocate for a paradigm shift in how we classify and approach the treatment of ovarian cancer. By moving beyond the constraints of histologic subtypes and delving into the molecular intricacies of patient-specific tumor microenvironments, we unveil a new opportunity for targeted immuno-oncological treatment of a subset of patients with ovarian cancer.

**Significance:**

Our study employs a multimodal latent variable model across 197 patients to identify principal drivers of immunogenomic intertumor heterogeneity. We uncover a distinct subgroup of endometrioid ovarian cancer with a unique immunogenomic signature and better prognosis and a set of collagen-related genes associated with the tumor microenvironment. This work challenges the adequacy of existing histologic classifications in capturing the molecular diversity of ovarian cancer, potentially informing more personalized treatment strategies.

## Introduction

Ovarian cancer is a gynecologic malignancy that ranks as the fourth most common cause of cancer-related death among women in the developed world (1). It is often (in roughly 70% of cases) diagnosed at an advanced stage when the disease has metastasized beyond the ovaries, making successful treatment more challenging. The overall 5-year survival rate for ovarian cancer is around 47%, emphasizing the urgent need for early detection and effective treatment options (2). The standard-of-care treatment regimen for ovarian cancer typically involves a multimodal approach, including surgery, chemotherapy, and, in some cases, targeted therapies. Malignant epithelial ovarian tumors, also known as carcinomas, are dominated by three major histologic subtypes–high-grade serous (HGSOC; 70%), endometrioid (10%), and clear cell (10%; ref. 3). Although specific genomic loci have been identified as the most common sites for somatic driver mutations or copy-number alterations (CNA) occurring in tumor-suppressive and oncogenes [including *TP53* (4, 5), *BRCA1/2* (6), *ARID1A* (7), *KRAS* (8), *PIK3CA* (9), *CTNNB1*, and *PTEN* (10, 11)], they do not explain the full breadth of heterogeneity observed in both the molecular makeup of tumors from different patients and the degree of penetration of their microenvironments by immune cells (12, 13).

Given the limited efficacy of immune checkpoint inhibitors in HGSOC and other ovarian cancer subtypes, they are often regarded as “immunologically cold” (14–16); however, such blanket statement does not necessarily apply to all ovarian cancers, and a more nuanced approach may be required for capturing the full breadth of variability across patients. Multiomics quantification of the molecular makeup of tumors allows for a deeper investigation of their phenotypes. However, extracting biologically interpretable insights from such datasets can be challenging because of the high dimensionality of each data modality relative to the number of samples, the statistical heterogeneity between different modalities, and the complex correlation structures between the profiled molecular entities. This necessitates the use of dimensional reduction techniques that can account for this. An ideal model would identify groups of molecular variables that feature interdependencies (and so can be inferred to participate in common biological processes) or, conversely, are orthogonal (independent) to one another. Multiomics factor analysis (MOFA; ref. 17) is a latent variable model that uses matrix factorization and Bayesian modeling to uncover orthogonal axes of heterogeneity in multimodal datasets. It can be intuitively understood as a generalization of principal component analysis (PCA) to multimodal data, and outputs results that can be interpreted in a similar manner. For example, in ref. 18, the authors used MOFA to jointly analyze DNA methylation, RNA sequencing, somatic mutation, and drug response data from blood derived from patients with chronic lymphocytic leukemia.

In this study, we present a cohort of 197 patients with ovarian cancer that we analyzed with MOFA applied to whole-exome sequencing (WES), transcriptomic quantification, and multiplex immunofluorescence (mIF) data to uncover the major axes of heterogeneity across HGSOC and less common histologic subtypes. We recovered previously known features of ovarian cancer, such as the mutually exclusive pattern of genomic alterations in *TP53* versus *ARID1A*, *KRAS*, *CTNNB1*, and *PIK3CA*, which explains differences between the histologic subtypes (HGSOC vs. others). We also identified novel immunogenomic profiles that transcend ovarian cancer subtypes. Most notably, however, our analysis reveals a distinct subgroup of endometrioid tumors characterized by markedly elevated *CST2* expression. This subgroup exhibits a unique tumor microenvironment with a reduced proliferation rate, enhanced MHC class II antigen presentation, and an associated improved prognosis. A deeper understanding of the immunogenomic landscape of ovarian cancer holds promise for the development of more effective therapies tailored to the unique molecular characteristics of ovarian cancer tumors.

## Materials and Methods

### Cohort summary

Surgical resections from primary malignant ovarian tumors were derived from 197 patients, with a median age of 60 years, a third of whom had a smoking history, with most diagnosed in stage III/grade 3 of the disease. The histopathologic classification of tumor subtypes was split into four major categories, dominated by HGSOC (62%), followed by endometrioid subtype (21%), and a small number of clear cell carcinoma (9%) and “other” subtypes; full details are listed in Supplementary Table S1. Most (96%) patients were treated with platinum therapy in the first line, to which most tumors (88%) were initially sensitive (Supplementary Table S1).

### Gene expression profiling

Formalin-fixed, paraffin-embedded (FFPE) tissue blocks from primary tumor resections were stained with hematoxylin and eosin for the purpose of marking up the tumor area and sent out for WES (protocol outlined below) to call somatic mutations (outsourced to Hologic). mRNA profiling was performed using the 770-gene human IO770 codeset and an AZ custom-designed 800-gene human myeloid codeset (Supplementary Table S2, NanoString Technologies) per NanoString’s recommended protocol, with 100 ng RNA input and including tumor reference and background controls (water). Raw count data were background-subtracted and housekeeping gene normalized in nSolver 4.0 (NanoString Technologies). Data obtained from different codeset manufacture lots were merged using CrossRLF and Batch Calibration Experiment tools in nSolver 4.0 and a final merging step was carried out to compare data from the two panels using the MultiRLF Merge Experiment tool in nSolver 4.0.

### WES protocol

DNA extraction of the FFPE sections with the Omega Bio-tek Mag-Bind FFPE DNA Kit was performed on a KingFisher Flex instrument. Extracted DNA was quantitated using the KAPA Human Genomic DNA Quantification and QC Kit and stored at −20°C. Whole-genome libraries were constructed using the KAPA HyperPlus Kit using a Beckman Coulter Biomek FxP liquid handler. Unique dual-indexed adapters containing a six base-pair unique molecular identifier (UMI) sequence, sourced from Integrated DNA Technologies, were ligated to the fragmented DNA. Libraries were quantitated using both the Agilent TapeStation D1000 assay and the KAPA Library Quantification Kit (qPCR). Hybridization capture was performed to enrich for the exonic, coding regions of the human genome. Before capture, whole-genome libraries were multiplexed, six samples per pool, with a total input of 2,000 ng per capture. The hybridization capture was performed manually using the Integrated DNA Technologies xGen Exome Research Panel v1.0 and the Roche NimbleGen Hybridization and Wash Kit. Whole-exome libraries were quantitated using both the Agilent TapeStation D1000 assay and the KAPA Library Quantification Kit (qPCR). Whole-exome libraries were sequenced using 2 × 150 chemistry on an Illumina HiSeq 4000 instrument.

Data were analyzed using pipeline software bcbio-nextgen v1.0.9 (https://doi.org/10.5281/zenodo.5781867). Reads were aligned to the hg38 reference using bwa v0.7.17, a quality control report was generated using multiqc, and sequencing duplicates for each UMI were collapsed into a single consensus read using fgbio. Samples were subsequently excluded from the analysis because of low quality as judged using average sequencing depth of coverage <50×, on-target reads <50%, and tumor purity ≤20% (see below). Variant calling was performed using VarDict v1.5.4 (19), down to a variant allele frequency (VAF) of 1% (before filtering and curation) and variant effects were annotated by snpEff v4.3t (20). Filtering of noncancer variants (i.e., common polymorphisms) was performed as per VarDict best practice. In addition, variants were removed if they satisfied any one of the following criteria: (i) VAF >95%, (ii) VAF <5%, (iii) <4 alternative reads supporting the variant, and (iv) variant depth <50. Potential artefacts induced by the fixing procedure were corrected using the DKFZ bias filter (https://github.com/DKFZ-ODCF/DKFZBiasFilter), such that SNVs seeming to be biased in terms of variant read support were removed.

### mIF profiling of immune cell populations within the tumor core and invasive margin

Sections (4 μm) were cut from FFPE tissue blocks, placed on slides, and assayed for IF for six immune markers in the order as listed: CD68 (macrophage marker; PG-M1 antibody from Agilent/Dako), PD-1 (D4W2J antibody from Cell Signaling Technology), PD-L1 (SP263 antibody from Ventana /Roche), CD8 (T-cell marker; SP239 antibody from Abcam), Ki67 (MIB-1 antibody from Agilent/Dako), and PANCK (AE1/AE3 antibody from Agilent/Dako) and finally stained with DAPI to facilitate image analysis. To account for the staining variability across batches, IF signal intensities were normalized (employing a min–max scaling procedure) using a positive tissue control stemming from primary ovarian cancer tumors, which was available for each batch of the assay run. The normalized images were coregistered with hematoxylin and eosin–stained slides from the same tissue block and annotated by a certified pathologist to select areas of the tumor core (TC) and the invasive margin (IM). The selected regions were then processed using an automated image analysis pipeline that determines, for each cell, its positivity (binary score) for each of the assayed markers. Cell-level readouts were aggregated to determine densities of cells positive for individual or multiple markers, separately in the TC and the IM areas of the tissue. Densities were measured in cells per mm^2^.

### Multiomics factor analysis

MOFA is a widely used statistical framework for dimension reduction in multimodal biomedical datasets (18). MOFA jointly models the different modalities of data to capture both shared and modality-specific sources of variation. Through matrix factorization and Bayesian modeling, MOFA identifies a small set of latent variables (called “factors” in MOFA terminology) that captures the variability across samples. These latent variables also come with weights (or “loadings”), akin to those outputs by a PCA, allowing for the easy biological interpretation of each latent variable.

The MOFA statistical framework was used for integrative analysis of the immunogenomic readouts available for the ovarian cancer sample set (18). By simultaneously analyzing data from different modalities, MOFA captures shared sources of variation, which are common across the different modalities, as well as the datatype-specific variability. Through matrix factorization, MOFA decomposes the multimodal dataset into a low-dimensional set of orthogonal latent variables (in MOFA terminology called “factors”) that, in descending order of percent variation, capture the underlying variability across samples, akin to the well-known PCA. Analogous to a PCA, MOFA outputs weights that indicate the contribution of each variable in the input data to each latent variable. By inspection of these weights, one can obtain a biological interpretation of each latent variable.

Somatic mutation data were available for two thirds of samples. Mutation calls were filtered to only retain genes which had at least five samples with driver mutations of confirmed clinical relevance as annotated by VarDict; variants of unknown significance were encoded as wild type. After filtering, mutation data from 16 unique genes were retained for fitting the MOFA model. NanoString mRNA abundance measures were available for 186 samples; data were filtered to only retain genes with the highest variance (≥1.5), which yielded 334 genes; values for each gene were then normalized using the Box–Cox transform. mIF data for the TC were available for all 197 samples but only for two thirds of samples for the area of the IM; 36 mIF density readouts (18 for TC and IM, each) were scaled to have unit variance. The MOFA+ framework allows for jointly modeling variation within samples belonging to different subgroups (19), which were taken to be the four major histopathologic categories listed in the Cohort Summary section. This setup aids in discerning between differences across samples that can be explained by the histologic subtype and those that occur universally across the cohort.

### Statistical analyses

Statistical analyses were performed in the R programming language. Differences between continuous values recorded across two groups of samples were tested for statistical significance using the Wilcoxon rank-sum test, whereas differences between categorical values across groups were evaluated using a Fisher exact test. Correlations were evaluated using the Spearman rank–based correlation coefficients. Mutual exclusivity of somatic mutations was evaluated using the Discrete Independence Statistic Controlling for Observations with Varying Event Rates (DISCOVER) framework (21). Hazard ratios between survival times across groups of samples were modeled using Cox proportional hazards models and statistical significance *P* values were obtained from the log-rank test. Principal component gene set enrichment analysis (PCGSEA; ref. 22) was used to associate the latent variables discovered using MOFA with biological pathways from the C2 set of 6,495 curated gene signatures available from MSigDB 7.0 by evaluating the mean difference set statistic (defined as the standardized difference between the mean of genomic variables in the gene set and genomic variables not in the set) over 10,000 permutations (23).

## Results

Drivers of intertumor heterogeneity, across all samples and within histologic subtypes, were uncovered using MOFA, which serves as a dimension reduction method for multimodal data. Following some initial variable selection, we input 386 immunogenic variables spanning three modalities into MOFA and samples from all the different subtypes were analyzed together. After manual inspection, we noted that the first 10 latent variables explained a significant portion of the overall variance while retaining biological interpretability. In total, these latent variables explained 9.0%, 51.2%, 86.9%, and 91.7% of variance in the somatic mutation, gene expression, mIF (TC), and mIF (IM) data modalities, respectively.

### Intertumor heterogeneity primarily driven by gene expression and immune context

**Figure 1.**
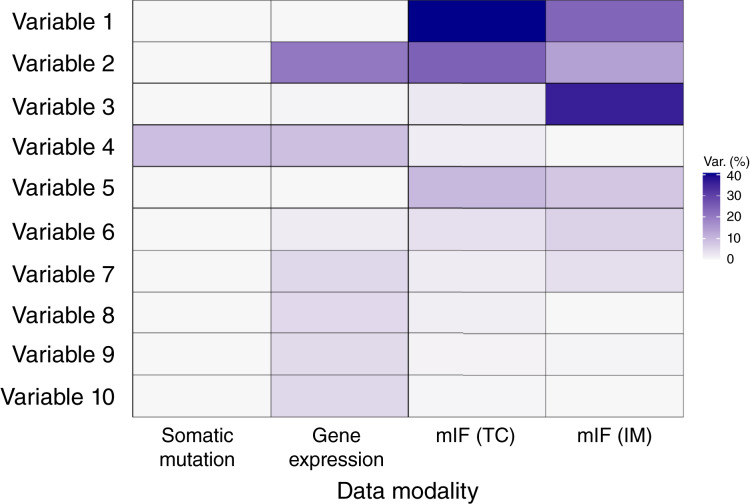
Percentage of variance explained in each modality by each of the MOFA-identified latent variables. Variable 1: Density of PD-1 and PD-L1 positivity in TC and IM; variable 2: expression of MHC-II antigen presentation pathway genes; variable 3: density of CD8^+^ and CD68^+^ cells in IM; variable 4: mutually exclusive mutations in tumors; variable 5: difference between density of CD8^+^ T cells and PD-L1+ tumor cells across tumor; variable 6: expression of specific chemokine-related genes; variable 7: expression of specific collagen-related genes; variable 8: expression of *CST2*; variable 9: expression of specific interferon-related genes; and variable 10: expression of *COL11A1*.

The strongest signals of immunogenomic intertumor heterogeneity in our ovarian cancer cohort came from the gene expression and the immune context (as measured by mIF) data, with patterns of somatic alterations explaining much less variability across the tumors (Fig. 1). Similar observations about the importance of gene expression over somatic mutation in driving intratumor heterogeneity are noted in colorectal cancer (24).

The majority of the variance in our samples was explained by the first 10 latent variables identified by MOFA. Each latent variable has associated “weights” that can be interpreted similarly to those output by a PCA. Thus, we can provide a biological characterization of these 10 axes of heterogeneity, which represent the 10 molecular quantities that in our cohort displayed the most variation across the tumors (Table 1).

**Table 1.** Summary of the 10 latent variables identified by MOFA as sources of heterogeneity in ovarian cancer^a^.

Variable	Description
Variable 1	Density of PD-1– and PD-L1–positive cells in the TC and IM.
Variable 2	Expression of genes in the MHC-II antigen presentation pathway inversely correlated with the density of proliferating tumor cells and positively correlated with the density of CD8^+^ T cells and CD68^+^ cells.
Variable 3	Density of CD8^+^ T cells and CD68^+^ cells in the IM.
Variable 4	Mutually exclusive mutations in tumors—*TP53* mutation mutually exclusive with mutations in the genes *ARID1A*, *KRAS*, *PIK3CA*, and *CTNNB1*.
Variable 5	The difference between the density of CD8^+^ T cells and PD-L1+ tumor cells across the tumor.
Variable 6	Expression of specific chemokine-related genes, in particular *CXCL8*, *CXCL1*, *CXCL3*, *CXCL6*, and *CCL5*.
Variable 7	Expression of specific collagen-related genes inversely correlated with the density of proliferating tumor cells.
Variable 8	Expression of *CST2*.
Variable 9	Expression of specific interferon-related genes, with pathway enrichment indicating a stronger association with *IFNα* and *IFNβ* than *IFNγ*.
Variable 10	Expression of *COL11A1*—adversely prognostic for both PFS and OS.

a These are the latent variables that explained at least 4% of variation in at least one modality in the data. Summaries were obtained by manual inspection of the variable weights, sometimes augmented by a PCGSEA (20), an adaptation of GSEA designed to work on feature weights.

### MHC-II activity inversely correlated with proliferating tumor cell density

**Figure 2.**
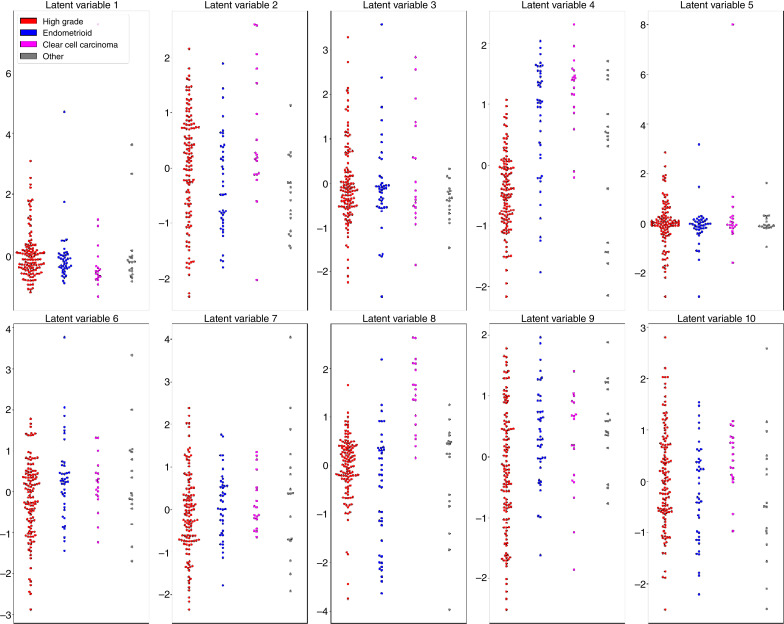
Beeswarm plots of the MOFA latent variables in each subtype. Variable 1: Density of PD-1 and PD-L1 positivity in TC and IM; variable 2: expression of MHC-II antigen presentation pathway genes; variable 3: density of CD8^+^ and CD68^+^ cells in IM; variable 4: mutually exclusive mutations in tumors; variable 5: difference between density of CD8^+^ T cells and PD-L1+ tumor cells across tumor; variable 6: expression of specific chemokine-related genes; variable 7: expression of specific collagen-related genes; variable 8: expression of *CST2*; variable 9: expression of specific interferon-related genes; and variable 10: expression of *COL11A1*.

Analysis of the weights for latent variable 2 highlighted that one of the most highly variable molecular quantities across the ovarian cancer samples was expression of genes in the MHC-II antigen presentation pathway (Fig. 1; Table 1) and this variation was independent of histologic subtype (Fig. 2).

**Figure 3.**
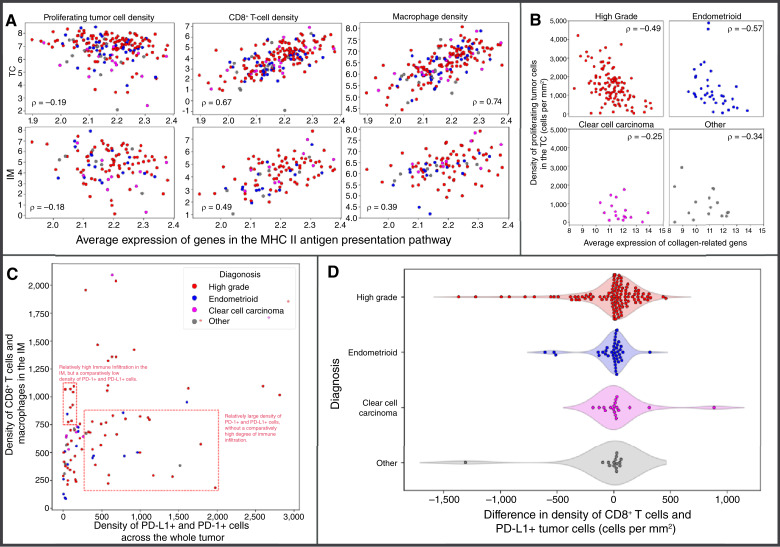
Immune cell features driving heterogeneity in ovarian cancer. **A,** Correlations between expression of genes in the MHC-II antigen presentation pathway and the density of proliferating tumor cells, CD8^+^ T cells, and macrophages in the TC and IM. All values plotted on a log scale. The coloring of points in this plot follows the scheme of the other panels. **B,** Scatter plots showing the relationship between the density of proliferating tumor cells and the expression of a group of collagen-related genes that were the highest weighted in MOFA latent variable 7 (*CCN2, COL1A1, CCN1, DCN, COL1A2, COL12A1, COL5A1, EGR1, COL3A1*, and *COL6A3*). **C,** Plot of T-cell inhibitor density and IM immune cell density. Dashed rectangles indicate groups of patients discussed in the main text. **D,** Beeswarm plot of the difference in density of CD8^+^ T cells and PD-L1+ tumor cells. A positive value means a higher density of PD-L1+ tumor cells than CD8^+^ T cells and *vice versa*.

Moreover, the average expression of genes in the MHC-II antigen presentation pathway was positively correlated with the density of T cells and macrophages and showed a very weak negative correlation with the density of proliferating tumor cells. In all cases, the correlations were stronger in the TC than in the IM (Fig. 3A).

### Specific collagen-related genes expressed by inflammatory fibroblasts are anticorrelated with densities of proliferating epithelial tumor cells

Latent variable 7 stood out by having high feature weight contributions from a set of collagen-related genes, including *CCN2, COL1A1, CCN1, DCN, COL1A2, COL12A1, COL5A1, EGR1, COL3A1,* and *COL6A3*, for which expression was inversely correlated with the density of proliferating tumor cells in the TC (Fig. 3B). This relationship was strongest in the endometrioid and HGSOC samples (Spearman rho ρ = −0.57; *P* = 8e−5, −0.5; 5e−9 respectively) and seemed weaker in the “Other” (ρ = −0.34; *P* = 0.2) and clear cell carcinoma samples (ρ = −0.25; *P* = 0.3).

Because stromal fibroblasts produce collagen to support tissue structure, and the tumor’s collagen-rich extracellular matrix undergoes substantial remodeling during tumor growth, it is possible that the collagen expression obtained by bulk mRNA quantification may be confounded by tumor purity (25). To assess this, we compared tumor purity, quantified by pathologist assessment with the average expression of the aforementioned collagen-related genes, and the two variables displayed negligible correlation (ρ = 0.09; *P* = 0.2 results not shown).

We then set out to identify the fibroblast type most likely to be responsible for the observed pattern of collagen-related gene expression. To this end, we used the collection of gene signatures characteristic of specific cancer-associated fibroblast (CAF) types from the pan-cancer single-cell resolution characterization study of CAFs (26). The available gene signatures allow distinguishing between eight major CAF subtypes, including two types of normal tissue–derived CAFs, cancer-associated myofibroblasts, inflammatory CAFs, adipogenic CAFs, endothelial-to-mesenchymal transition CAFs, peripheral nerve–like CAFs, and antigen-presenting CAFs. PCGSEA using the weights for genes from latent variable 7 showed the strongest enrichment for signatures indicative of inflammatory CAFs (*P* < 0.001) and weaker enrichment for two clusters of normal tissue–derived CAFs (*P* = 0.001 and 0.002, respectively). The decreased density of proliferating cells (identified by Ki67 positivity) in tumors that have a higher concentration of inflammatory CAFs (Supplementary Fig. S1) aligns with their earlier characterization as having relatively low proliferation rates (26).

Analysis of latent variable 10, which is independent from latent variable 7, identified *COL11A1* as one of the top contributing genes. In a separate survival analysis, we found that higher expression of *COL11A1* was significantly associated with worse overall survival (OS) and progression-free survival [PFS; *COL11A1* high vs. *COL11A1* low: HR = 1.16; 1.04–1.29; log-rank test *P* value = 0.003–0.008, Supplementary Fig. S2]. *COL11A1 *expression did not display any significant relationship with the density of proliferating tumor cells, and none of the other collagen genes mentioned above were significantly associated with OS or PFS.

### Density of immune cells in the tumor microenvironment as a source of heterogeneity in ovarian cancer

The density of PD-1- or PD-L1–positive cells (the highest weighted variables in latent variable 1) accounted for a large proportion of variability across patients with ovarian cancer—latent variable 1 explained 41% of variation in the TC and 24% in the IM data. It also displays a degree of independence from the density of T cells and macrophages in the IM (the highest weighted variables in latent variable 3). This is illustrated in Fig. 3C, in which despite some correlation between the two molecular quantities, there is a distinct subset of patients with a high density of CD8 T cells and macrophages in the IM, whereas the density of PD-1/PD-L1+ cells across the tumor remains relatively low. This effect was unique to the IM; in the TC, the density of CD8 T cells and macrophages and the density of PD-1/PD-L1 were well correlated (Supplementary Fig. S3). Notably, the density of T cells and macrophages in the TC and in the IM was also well correlated across ovarian cancer samples. Conversely, there was also a distinct subgroup of patients with a comparatively low density of CD8 T cells and macrophages in the IM but a relatively high density of PD-1/PD-L1 across the tumor. In these patients, there was limited recruitment of immune cells to the tumor despite the high levels of immune checkpoint inhibitors (Fig. 3C).

We looked at the difference in the density of CD8^+^ T cells and PD-L1+ tumor cells as a proxy for quantifying the effectiveness of the immune response. We observed that within the HGSOC samples, the distribution exhibited a pronounced heavy tail at the lower end, a feature not observed in the endometrioid or clear cell carcinoma samples (Fig. 3D). This highlights that HGSOC samples more often display extreme levels of CD8^+^ T cells or PD-L1+ T-cell inhibitors in comparison with endometrioid ovarian cancers and clear cell carcinomas, which exhibit a comparatively narrow range of T-cell and checkpoint inhibitor levels.

### Mutually exclusive mutations

Inspection of features with high weight contributions to latent variable 4 recovered the well-known pattern of mutually exclusive mutations in *TP53* (27), which was mutant predominantly in the HGSOC samples, versus *ARID1A, KRAS, PIK3CA*, and *CTNNB1*, which were primarily mutant in endometrioid ovarian cancer samples [significance evaluated using the DISCOVER test (19), *P* = 0.007]. This reflects the classic pattern of key driver mutations in ovarian cancer.

Latent variable 4 also explained some variation in the gene expression data (Fig. 1). We observed that HGSOC samples feature elevated expression levels of interferon signaling genes (according to Reactome pathway analysis) compared with the other subtypes (HGSOC against all other subtypes combined *P* = 0.0004, Supplementary Fig. S4). However, this effect may be confounded by *TP53 *status given the high prevalence of *TP53* mutations in patients with HGSOC as *TP53*-mutant samples also featured elevated expression levels of genes in the same pathway (*P* = 0.0007, Supplementary Fig. S5).

### Immunogenomic variability across ovarian cancer samples cannot be explained by histologic subtype alone

Most of the latent variables did not show an association with disease subtype; indeed, for many of them, the distribution of their values is very similar within each histologic classification (Fig. 2). This indicates that many of the main drivers of immunogenomic intertumor heterogeneity in ovarian cancer are not accounted for by histologic subtype, rather they display significant variation within each subtype.

Tumors of all subtypes displayed substantial variation in patterns of immune cell activation as evidenced by PD-1/PD-L1 and CD8/CD68 cell densities (latent variables 1, 3). Furthermore, the expression of genes in the MHC-II antigen presentation pathway (as determined by Reactome pathway analysis) was highly variable across all tumor types and did not display any significant relationship with disease subtype (latent variable 2). Despite the general distributional similarities across histotypes, most of the latent variables exhibited higher variation within the HGSOC samples, indicating that this is a more immunogenomically unstable subtype.

**Figure 4.**
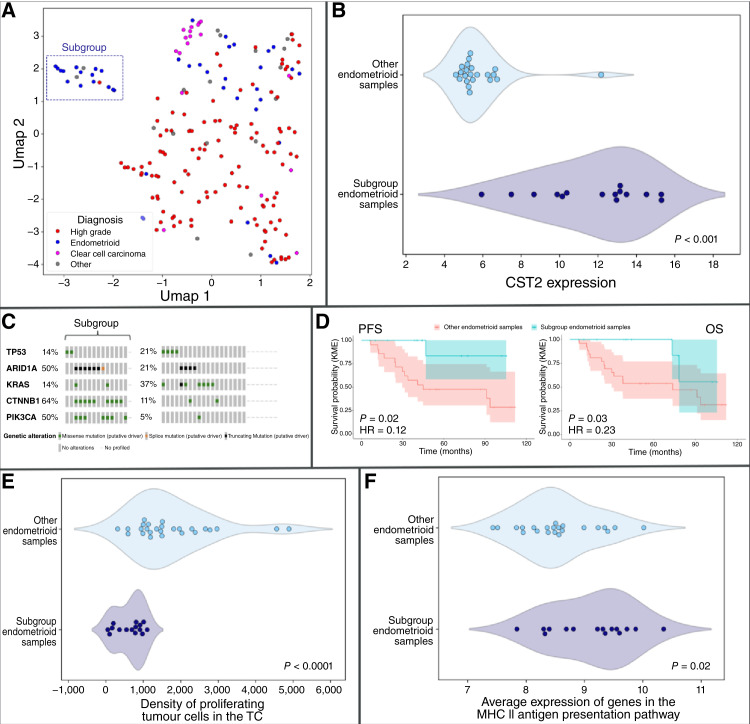
Plots detailing the subgroup of endometrioid samples. **A,** Uniform Manifold Approximation and Projection (UMAP) plot of the samples according to their position in latent variable space, showing clearly the subgroup of endometrioid patients discussed in the main text. **B,** Expression of *CST2* for endometrioid samples stratified by subgroup membership. **C,** Oncoprint of selected genes for endometrioid samples stratified by subgroup membership. **D,** Survival curves for endometrioid samples stratified by subgroup membership. **E,** Density of proliferating tumor cells and (**F**) MHC-II antigen presentation expression for endometrioid samples stratified by subgroup membership.

The only molecular quantities that displayed significant relationships with disease subtype were *TP53* mutation, which as is well known was associated with HGSOC, and *CST2 *expression; a subset of the endometrioid ovarian cancer samples exhibited elevated expression of *CST2 *(Fig. 4B, *P* < 0.0001), and all clear cell carcinoma samples exhibited very low expression of *CST2*.

### Uniform Manifold Approximation and Projection analysis reveals a molecularly distinct subgroup of endometrioid patients

After using MOFA to identify the primary immunogenomic drivers of intertumor heterogeneity in our cohort, we wanted to investigate whether samples clustered according to any of these drivers.

Figure 4A displays a Uniform Manifold Approximation and Projection plot of the samples in two dimensions, with the projection based on the values of the MOFA-identified latent variables for each sample (28). There is a visually distinct cluster consisting primarily of endometrioid ovarian cancer samples, disjoint from the remaining samples included in the cohort. We referenced the latent variables to determine whether the endometrioid samples in this subgroup (*n* = 15) differed significantly in any biologically meaningful ways from the other endometrioid samples (*n* = 23). There were no significant differences in age, stage, or grade between the patients in the two endometrioid cancer groups (Supplementary Fig. S6). To ensure that we are not just picking up on characteristics of endometrioid ovarian cancer, all comparisons in the rest of this section are between the endometrioid ovarian cancer samples in the subgroup and the rest of the endometrioid ovarian cancer samples.

Most strikingly, the tumors in the subgroup exhibited marked overexpression of the cystatin SA (*CST2*) gene (*P* < 0.0001; Fig. 4B) and featured significantly elevated rates of somatic driver mutations in hallmark oncogenes *CTNNB1* and *PIK3CA *(Fisher exact test *P* = 0.003 and *P* = 0.005, respectively, Fig. 4C) and did not harbor mutations in genes causing homologous repair deficiency (homologous repair deficiency–associated genes we considered included *BRCA1/2, ATM, BARD1, BRIP1, CDK12, CHEK1/2, FANCL, PALB2, PPP2R2A, RAD51B/C/D*, and *RAD5*; *t* test *P* value = 0.04). Moreover, the subgroup samples display markedly reduced densities of proliferating tumor cells in the TC (as indicated by the densities of CK+, KI67+ double-positive cells, Wilcoxon rank-sum test *P* value = 4 × 10e−6, Fig. 4E), which was correlated with a moderate increase in the expression of genes involved in the MHC-II antigen presentation pathway [according to Reactome annotations (29), Wilcoxon rank-sum test *P* value = 0.02, Fig. 4F]. Additionally, we performed pathway enrichment analysis and confirmed that Wnt–β-catenin signaling is the top hallmark associated with the *CST2*-high subset of patients with endometrioid ovarian cancer in our cohort (normalized enrichment score = 1.7; *P* = 0.01). Finally, the patients in the subgroup had a better prognosis for both OS and PFS (HR, 0.12–0.23, log-rank test *P* values, 0.02–0.03, respectively, Fig. 4D) as one might expect given the elevated levels of MHC-II activity and reduced density of proliferating tumor cells.

To validate the above findings, we identified a subset of 51 endometrioid ovarian cancer samples within the TEMPUS Real World Evidence dataset we have access to. We have used batch-corrected transcripts per million values to split the patient population according to *CST2* expression, defining 27 samples as *CST2*-high and 24 as *CST2*-low expressors. The enrichment of *CTNNB1* mutations of known or likely clinical significance was significantly higher in the *CST2*-high subgroup (52% vs. 9%, Fisher exact test *P* value = 0.0009) but we have not observed any difference in the *PIK3CA* driver mutation prevalence (Fisher exact test *P* value ≈ 1). Bulk RNA deconvolution using xcell (30) indicated a higher ImmuneScore for the *CST2*-high samples (Wilcoxon test *P* value = 0.02), as well as a trend (Wilcoxon test *P* value = 0.06) for higher MHC class II antigen presentation activity, based on a comparison of MHC class II gene expression signature activity (using the gene set REACTOME_MHC_CLASS_II_ANTIGEN_PRESENTATION) in each sample, obtained via single-sample gene set variation analysis (31). Because of survival data immaturity (events recorded for only six of 51 patients), outcome analysis could not be performed.

## Discussion

In this study, we attempted to uncover sources of molecular heterogeneity among 197 patients with ovarian cancer (HGSOC: 122, endometrioid: 42, clear cell: 18, and other: 16). We found that immunogenomic traits are the main drivers of intertumor heterogeneity using a robust factor analysis model that integrates multiomic datasets in an unsupervised fashion. We described 10 major axes of heterogeneity that demonstrated the highest variation among patients with ovarian cancer. Heterogeneity sources included expression of PD-1 and PD-L1 in the TC and IM, expression of the MHC-II antigen presentation pathway genes, density of CD8^+^ T cells and CD68^+^ macrophages in the IM, genetic mutations, expression of specific chemokine, collagen and interferon genes, and expression of the *CST2* and *COL11A1* genes.

One of the greatest sources of molecular heterogeneity among patients with ovarian cancer stemmed from the immune contexture and PD-1/PD-L1 positivity, primarily in the TC but also in the IM, indicating variable immune activity within and across different histologic subtypes in ovarian cancer. The highest variation was observed in patients with HGSOC with a subset of those displaying high densities of immune cells and checkpoint molecules, suggesting that these patients may be more amenable to immunotherapy than other HGSOC or other subtype patients; clinical trials would further elucidate whether the high immune cell content in this subset of patients could warrant better therapeutic response to ICIs. Interestingly, we observed two groups of HGSOC samples with distinct immune microenvironment characteristics; a group of PD-1–/PD-L1–high expressors with low CD8^+^/CD68^+^ cell infiltration in the IM and a second group with the opposite relationship, indicating that PD-1/PD-L1 expression alone may be insufficient for predicting the ability to mount an inflammatory response and immunotherapeutic efficacy.

Antitumor immunity delivered by the MHC-II antigen presentation pathway was highly variable in all histologic subtypes, reflecting discrepancies in immune responses against tumor growth among patients with ovarian cancer. MHC-II gene expression was positively correlated with CD8^+^ T-cell and CD68^+^ macrophage recruitment to tumor site and inversely correlated with tumor proliferation dynamics. Similarly, other studies have reported that tumors with higher MHC class II pathway activity exhibited lower growth rates in ovarian cancer (32), highlighting potential anticancer therapeutic benefit through MHC-II pathway manipulation.

Furthermore, we showed that the expression of collagen-related genes and the *COL11A1* gene represent a significant source of molecular heterogeneity in ovarian cancer. We found that specific collagen-related genes expressed by inflammatory fibroblasts are anticorrelated with proliferation of epithelial tumor cells and *COL11A1* gene is associated with poor PFS and OS. Previous studies of pancreatic ductal adenocarcinoma have indicated that the presence of CAFs may be indicative of tumors exhibiting an immunosuppressive phenotype (33), whereas others linked the poor survival outcomes in ovarian cancer to the role of *COL11A1* in activating the ERK pathway and the TGFb1–MMP3 axis (34, 35). Overexpression of *COL11A1* has been recently suggested as a biomarker of poor outcomes across multiple solid tumors, including ovarian, breast, gastric, head and neck, pancreatic, colorectal, glioma, and salivary gland cancers (36).

Interestingly, for most variables, intertumor variability could not be explained by histologic subtype alone as data distribution across histotypes was similar, while displaying substantial variation within each histotype. However, variable 4 which pertained to somatic mutation landscape did differ between histologic types, confirming the already established pattern of mutual exclusivity of *TP53* mutations, predominantly present in patients with HGSOC, and *ARID1A, KRAS, PIK3CA*, and *CTNNB1* mutations, primarily encountered in endometrioid ovarian cancer (27). A substantial amount of variation with regard to variable 4 was also explained by higher interferon gene expression in HGSOC samples compared with other subtypes (*P* value = 0.0004). However, this effect may be confounded by *TP53* status given the high prevalence of this mutation in patients with HGSOC. Taken together, all *TP53*-mutated samples exhibit a strong signal for elevated expression of interferon-related genes (*P* value = 0.0007), which is in agreement with previous studies investigating the effects of *TP53* somatic mutations across various malignant indications (37, 38).

Cystatin SA (*CST2*) expression was another variable that differed according to histologic subtype, with a subset of endometrioid ovarian cancer samples harboring *PIK3CA* and *CTNNB1* mutations, featuring high levels of *CST2* expression, low tumor cell proliferation markers in TC, moderate increase in MHC-II expression, and favorable PFS and OS. Interestingly, although it is not regarded as a hallmark oncogene (*CST2* is not featured in tier 1 or 2 of the Catalogue of Somatic Mutations in Cancer Gene Census; ref. 11), *CST2* has previously been implicated as a key gene driving the breast cancer phenotype, exhibiting a 350-fold increase in the mRNA expression measured in the tumor compared with the adjacent paracancerous tissue (39). Other studies have observed that patients with high *CST2* expression had a better prognosis in gastric cancer (40), and with longer time to recurrence after neoadjuvant chemotherapy treatment in HGSOC (41). It is also noteworthy that a previous analysis of endometroid ovarian carcinomas by Hollis and colleagues (42) subcategorized cases into three genomic classes according to their *CTNNB1* and *TP53* mutation status (*TP53*mut, *CTNNB1*wt/*TP53*wt, *CTNNB1*mut/*TP53*wt, and *CTNNB1*wt) and reported favorable outcomes for the *CTNNB1*mut/*TP53*wt group. That study, however, lacked the additional immuno-transcriptomic profiling that allowed implicating *CST2* activity in driving this phenotype. Using pathway enrichment analysis, we confirmed that Wnt–β-catenin signaling is the top hallmark associated with the *CST2*-high subset of patients with endometrioid ovarian cancer in our cohort. This is consistent with Wang and colleagues (43) analysis of *CST2* expression in the context of serous ovarian carcinoma. Their analysis indicated that *CST2* activates Wnt–β-catenin signaling and is associated with higher immune cell infiltration but is associated with an unfavorable prognosis. The differential impact of *CST2* expression on patient outcomes in endometrioid versus serous ovarian cancer highlights the complexity of cancer biology and the importance of immunogenomic context in the tumor microenvironment. Taken together, these observations might reveal a new druggable target (https://platform.opentargets.org/target/ENSG00000170369) in a subset of patients with ovarian cancer or a prognostic biomarker in relation to survival status.

Notably, although CNAs are known to play a significant role in the development and progression of ovarian cancer, the categorical call data on CNAs we had available did not enrich the latent variables discovered by MOFA; hence, they were excluded from analysis. This represents a limitation of this study, and we acknowledge that good-quality CNA data could provide complementary information. Furthermore, another caveat is that the percentage of variance presented refers only to the data retained after the initial prefiltering steps as the variants of unknown significance, as well as the less variable transcriptomic and immune features, were disregarded from analysis. Lastly, novel single-cell and spatial technologies (e.g., single-cell RNA sequencing, single-cell assay for transposase-accessible chromatin using sequencing, Visium, and Xenium) could enhance subcellular and spatial context resolution and allow for detailed mapping of the tumor microenvironment, highlighting additional sources of heterogeneity in ovarian cancer.

### Conclusion

Although ovarian cancer tumors are traditionally considered “immune cold” and immune checkpoint blockade has so far demonstrated limited efficacy in the ovarian cancer setting, our analysis reveals that there is a substantial amount of variation in the immunogenomic make up of ovarian cancer tumors, indicating that such a blanket view neglects the underlying heterogeneity of ovarian cancer. Moreover, our analysis indicates that this variation cannot be accounted for by histologic subtype alone. Thus, selection criteria for choosing the optimal treatment options for patients with ovarian cancer should extend beyond histologic types and include individual immunogenomic traits for each patient to identify that a subset of patients, however small, that could potentially derive benefit from immunotherapeutic regimens based on prospective biomarker validation studies.

## Supplementary Material

Supplementary Table 1 Patient demographics including age, histological subtype, grade, stage, radiation or platinum therapy, platinum resistance, HRD and BRCA status.

Supplementary Figure 1 Relationship between the density of proliferating (KI67+) cells and the average expression of genes found to characterise inflammatory CAFs in [24]

Supplementary Table 2 Nanostring gene panel

Supplementary Figure 2 Overall survival (a) and progression-free survival (b) curves for patients stratified by COLL1A1 expression.

Supplementary Figure 3 Plots of T Cell Inhibitor versus Immune Cell density across different regions of the tumour.

Supplementary Figure 4 Expression of interferon signalling genes in different ovarian cancer subtypes, p-value is comparing the high-grade distribution against all other subtypes combined.

Supplementary Figure 5 Expression of interferon signalling genes in TP53 mutant and wild type samples.

Supplementary Figure 6 Age (a), stage (b) and grade (c) for the two endometrioid patient subgroups identified in Figure 4a.

## Data Availability

Data underlying the findings described in this article may be obtained in accordance with AstraZeneca’s data-sharing policy described at https://astrazenecagrouptrials.pharmacm.com/ST/Submission/Disclosure. Requests to access the data described in the current article can be submitted through https://vivli.org/members/enquiries-about-studies-not-listed-on-the-vivli-platform/. Tempus-derived deidentified data used in this research were collected in a real-world health care setting and are subject to controlled access for privacy and proprietary reasons. When possible, derived data supporting the findings of this study have been made available within the article and its Supplementary Figures/Tables. Restrictions apply to the availability of additional data, which were used under license for this study.
