# Supplementary material for: Characterization of the Immunogenomic Landscape of Ovarian Cancer Uncovers a Distinct Subset of Endometroid Tumors Associated with High CST2 Expression and a Favorable Prognosis
Source: Cancer Res Commun. 2026 Jan 28;6(1):224–34. doi: 10.1158/2767-9764.CRC-25-0150 (PMC12848861; doi:10.1158/2767-9764.CRC-25-0150)
Supplement: Supplementary Table 1 — Patient demographics including age, histological subtype, grade, stage, radiation or platinum therapy, platinum resistance, HRD and BRCA status. [file crc-25-0150_supplementary_table_1_suppst1.pptx]

## Slide 1
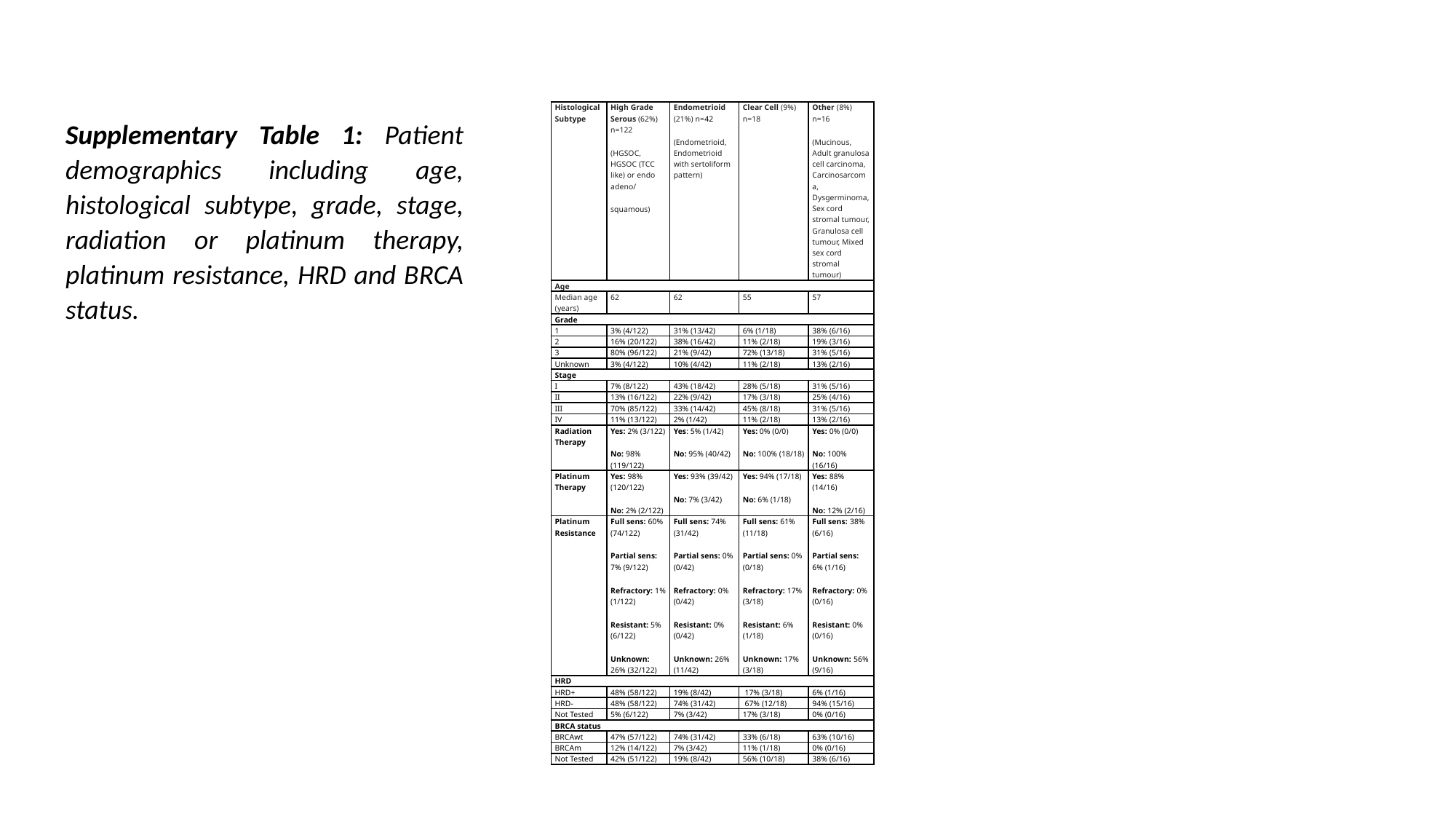

| Histological Subtype | High Grade Serous (62%) n=122 (HGSOC, HGSOC (TCC like) or endo adeno/ squamous) | Endometrioid (21%) n=42 (Endometrioid, Endometrioid with sertoliform pattern) | Clear Cell (9%) n=18 | Other (8%) n=16 (Mucinous, Adult granulosa cell carcinoma, Carcinosarcoma, Dysgerminoma, Sex cord stromal tumour, Granulosa cell tumour, Mixed sex cord stromal tumour) |
| --- | --- | --- | --- | --- |
| Age | | | | |
| Median age (years) | 62 | 62 | 55 | 57 |
| Grade | | | | |
| 1 | 3% (4/122) | 31% (13/42) | 6% (1/18) | 38% (6/16) |
| 2 | 16% (20/122) | 38% (16/42) | 11% (2/18) | 19% (3/16) |
| 3 | 80% (96/122) | 21% (9/42) | 72% (13/18) | 31% (5/16) |
| Unknown | 3% (4/122) | 10% (4/42) | 11% (2/18) | 13% (2/16) |
| Stage | | | | |
| I | 7% (8/122) | 43% (18/42) | 28% (5/18) | 31% (5/16) |
| II | 13% (16/122) | 22% (9/42) | 17% (3/18) | 25% (4/16) |
| III | 70% (85/122) | 33% (14/42) | 45% (8/18) | 31% (5/16) |
| IV | 11% (13/122) | 2% (1/42) | 11% (2/18) | 13% (2/16) |
| Radiation Therapy | Yes: 2% (3/122) No: 98% (119/122) | Yes: 5% (1/42) No: 95% (40/42) | Yes: 0% (0/0) No: 100% (18/18) | Yes: 0% (0/0) No: 100% (16/16) |
| Platinum Therapy | Yes: 98% (120/122) No: 2% (2/122) | Yes: 93% (39/42) No: 7% (3/42) | Yes: 94% (17/18) No: 6% (1/18) | Yes: 88% (14/16) No: 12% (2/16) |
| Platinum Resistance | Full sens: 60% (74/122) Partial sens: 7% (9/122) Refractory: 1% (1/122) Resistant: 5% (6/122) Unknown: 26% (32/122) | Full sens: 74% (31/42) Partial sens: 0% (0/42) Refractory: 0% (0/42) Resistant: 0% (0/42) Unknown: 26% (11/42) | Full sens: 61% (11/18) Partial sens: 0% (0/18) Refractory: 17% (3/18) Resistant: 6% (1/18) Unknown: 17% (3/18) | Full sens: 38% (6/16) Partial sens: 6% (1/16) Refractory: 0% (0/16) Resistant: 0% (0/16) Unknown: 56% (9/16) |
| HRD | | | | |
| HRD+ | 48% (58/122) | 19% (8/42) | 17% (3/18) | 6% (1/16) |
| HRD- | 48% (58/122) | 74% (31/42) | 67% (12/18) | 94% (15/16) |
| Not Tested | 5% (6/122) | 7% (3/42) | 17% (3/18) | 0% (0/16) |
| BRCA status | | | | |
| BRCAwt | 47% (57/122) | 74% (31/42) | 33% (6/18) | 63% (10/16) |
| BRCAm | 12% (14/122) | 7% (3/42) | 11% (1/18) | 0% (0/16) |
| Not Tested | 42% (51/122) | 19% (8/42) | 56% (10/18) | 38% (6/16) |
Supplementary Table 1: Patient demographics including age, histological subtype, grade, stage, radiation or platinum therapy, platinum resistance, HRD and BRCA status.
