# Supplementary material for: Characterization of the Immunogenomic Landscape of Ovarian Cancer Uncovers a Distinct Subset of Endometroid Tumors Associated with High CST2 Expression and a Favorable Prognosis
Source: Cancer Res Commun. 2026 Jan 28;6(1):224–34. doi: 10.1158/2767-9764.CRC-25-0150 (PMC12848861; doi:10.1158/2767-9764.CRC-25-0150)
Supplement: Supplementary Figure 1 — Relationship between the density of proliferating (KI67+) cells and the average expression of genes found to characterise inflammatory CAFs in [24] [file crc-25-0150_supplementary_figure_1_suppsf1.pptx]

## Slide 1
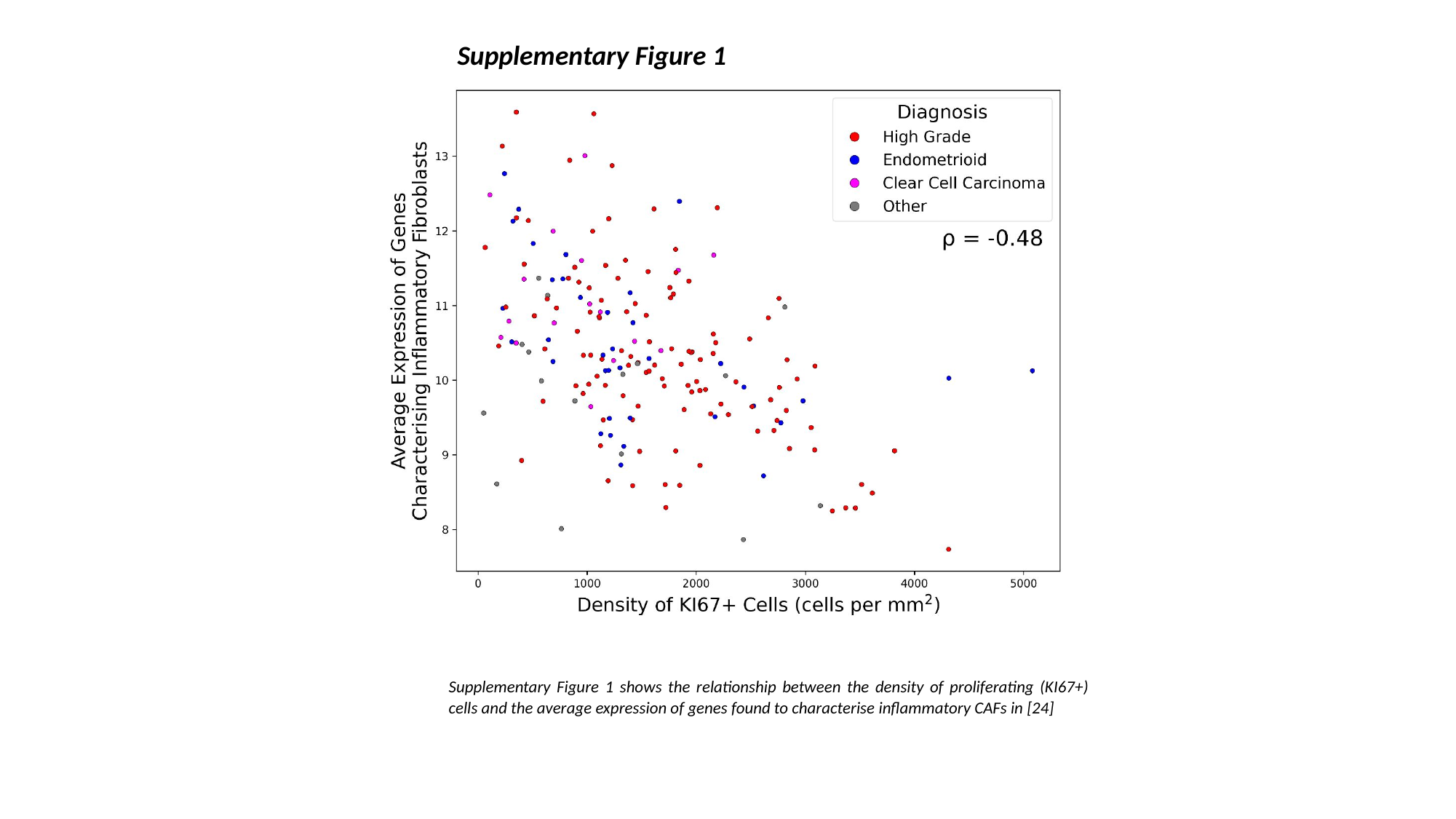

Supplementary Figure 1
Supplementary Figure 1 shows the relationship between the density of proliferating (KI67+) cells and the average expression of genes found to characterise inflammatory CAFs in [24]
