# Supplementary material for: Characterization of the Immunogenomic Landscape of Ovarian Cancer Uncovers a Distinct Subset of Endometroid Tumors Associated with High CST2 Expression and a Favorable Prognosis
Source: Cancer Res Commun. 2026 Jan 28;6(1):224–34. doi: 10.1158/2767-9764.CRC-25-0150 (PMC12848861; doi:10.1158/2767-9764.CRC-25-0150)
Supplement: Supplementary Table 2 — Nanostring gene panel [file crc-25-0150_supplementary_table_2_suppst2.pptx]

## Slide 1
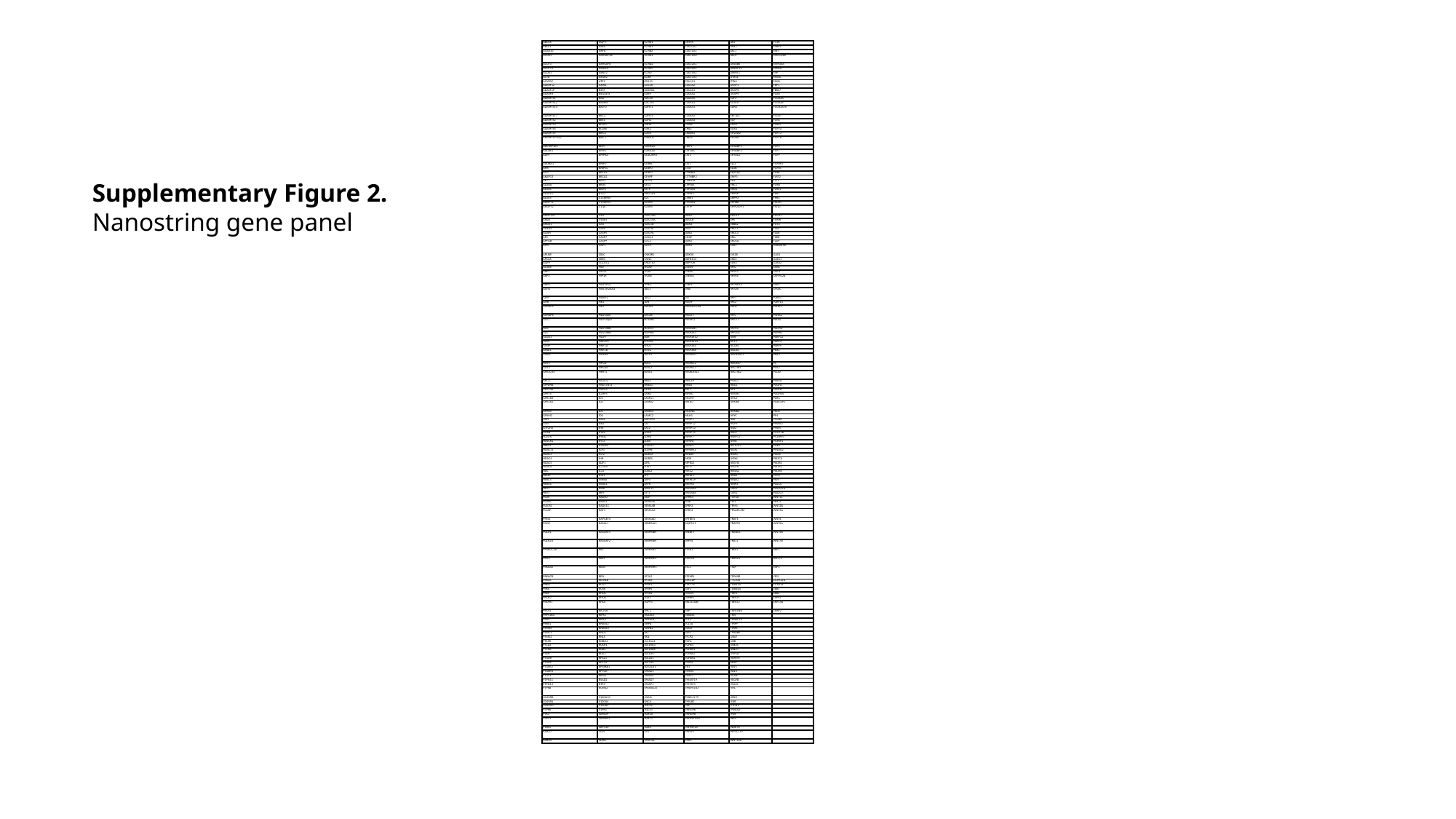

| ABCC8 | AQP9 | CCNA1 | CNTFR | DES | F11R |
| --- | --- | --- | --- | --- | --- |
| ABCF1 | AREG | CCNB1 | COL10A1 | DKK1 | FABP4 |
| ACAD10 | ARF6 | CCNB2 | COL11A1 | DLL1 | FAF1 |
| ACOD1 | ARHGEF28 | CCND1 | COL11A2 | DLL4 | FAM124B |
| ACOT1 | ARHGEF6 | CCND2 | COL12A1 | DNAJB6 | FAM30A |
| ACOT11 | ARID1A | CCNE1 | COL14A1 | DNAJC14 | FANCA |
| ACOX1 | ARNT2 | CCNO | COL15A1 | DNMT1 | FAP |
| ACTB | ASGR2 | CCR8 | COL17A1 | DTX3L | FASLG |
| ACVR1C | ATF3 | CD151 | COL1A1 | DTX4 | FASN |
| ADAM12 | AXIN1 | CD226 | COL1A2 | DUSP1 | FBP1 |
| ADAM19 | B2M | CD300A | COL4A1 | DUSP2 | FBXL7 |
| ADAM8 | B4GALT4 | CD69 | COL4A2 | DUSP5 | FCAR |
| ADAMTS1 | BAD | CDC20 | COL4A5 | E2F3 | FCGR3A |
| ADAMTS12 | BAMBI | CDC25C | COL5A1 | ECSCR | FCGR3B |
| ADAMTS14 | BATF3 | CDH11 | COL6A1 | EDN1 | FCGR3A/B |
| ADAMTS17 | BBC3 | CDH13 | COL6A2 | EEF1A1 | FCGRT |
| ADAMTS2 | BBS1 | CDH2 | COL6A3 | EGF | FCN1 |
| ADAMTS3 | BCAT1 | CDH4 | COMP | EGFR | FCRL2 |
| ADAMTS4 | BCL6B | CDK2 | CPA3 | EGR3 | FGF10 |
| ADAMTS9 | BIRC2 | CDK6 | CRABP2 | EIF2AK2 | FGF13 |
| ADAMTS9-AS2 | BIRC3 | CDKN1C | CREM | EIF2B4 | FGF18 |
| ADCYAP1R1 | BLM | CDKN2A | CRIP1 | EIF4EBP1 | FGF2 |
| ADGRE1 | BMP2 | CDKN2B | CSF2RA | EIF4EBP2 | FGF7 |
| ADM | BMP8A | CEACAM3 | CST2 | EIF5AL1 | FGF9 |
| ADORA3 | BNIP3 | CEBPA | CST7 | ELL2 | FGFBP2 |
| AHR | BNIP3L | CEBPD | CTGF | ELOB | FGFR1 |
| AIF1 | BRCA1 | CEBPG | CTNNB1 | ELOVL6 | FLNB |
| AKAP13 | BRCA2 | CENPF | CTTNBP2 | EMP1 | FLRT2 |
| AKT1 | BRD3 | CEP55 | CXorf36 | EN1 | FLT1 |
| ALDOA | BRD4 | CES3 | CYP1B1 | ENC1 | FOSB |
| ALDOC | BRIP1 | CETP | CYP3A4 | ENO1 | FOSL1 |
| ALOX15 | BTG2 | MB21D1 | CYP4F3 | ENPEP | FPR1 |
| ALOX5 | C11orf45 | CLC | CYR61 | ENPP2 | FPR3 |
| ANGPT1 | C15orf53 | CLDN1 | CYSLTR1 | EPHB6 | FSCN1 |
| ANGPT2 | C1QC | CLDN5 | CYTIP | EPM2AIP1 | FSTL3 |
| ANGPTL4 | C4A | CLEC10A | DAB2 | EPSTI1 | FUCA1 |
| ANLN | C5AR1 | CLEC14A | DAGLB | EPX | FURIN |
| ANOS1 | CA4 | CLEC1B | DAXX | ERBB2 | FUT4 |
| ANXA4 | CALR | CLEC4E | DCN | ERCC1 | FZD4 |
| AOAH | CASP5 | CLEC9A | DDB2 | ERCC3 | FZD6 |
| APC | CASP7 | CLECL1 | CHOP | ERG | FZD8 |
| APH1B | CASP9 | CLIC2 | DDR2 | ERO1A | FZD9 |
| API5 | CAV1 | CLIC4 | DDX4 | ESR1 | GADD45B |
| APLNR | CBLC | CNKSR1 | DDX50 | EVI2B | GAS1 |
| APOL6 | CBR1 | CNN2 | DEFB134 | EXO1 | GATA1 |
| AQP9 | CCL3/L1 | CNOT10 | DEPTOR | EZH2 | GATA2 |
| GATA6 | HGF | ITGAV | LTA4H | MSC | OASL |
| GBP1 | HIF1A | ITGB7 | LTB4R | MSH2 | OAZ1 |
| GBP2 | HIP1R | ITGB8 | LTB4R2 | MSH6 | OLFML2B |
| GBP4 | HIST1H1C | ITPK1 | LTBP1 | MTMR14 | OLR1 |
| GCH1 | HIST2H2AA3 | JAG1 | LY6E | MTOR | OTOA |
| GEM | HIVEP1 | JAG2 | LYZ | MPT | P2RX1 |
| GHR | HK1 | JUN | MAFF | MX2 | P2RY13 |
| GIMAP4 | HK2 | KALRN | MAGEA3/A6 | MXI1 | P4HA1 |
| GIMAP6 | HLA-DOA | KAT2B | MALT1 | MYC | P4HA2 |
| GLG1 | HLA-DQA2 | KCNAB1 | MAML2 | MYCT1 | PADI4 |
| GLI1 | HLA-DRB1 | KCNJ15 | MAN2B1 | MYH4 | PALMD |
| GLS | HLA-DRB5 | KDM6B | MAP2K3 | MYOD1 | PAMR1 |
| GLUD1 | HLA-F | KDR | MAP3K12 | NBN | PARP12 |
| GLUL | HMGA1 | KIF20A | MAP3K14 | NCF2 | PARP4 |
| GMIP | HNF1A | KIF2C | MAP3K4 | NCOR1 | PARP9 |
| GNAI3 | HNF1B | KITLG | MAP3K8 | NDC80 | PBX1 |
| GNG4 | HOXD4 | KLF10 | MAPK10 | NDUFA4L2 | PBX3 |
| GOT1 | HPGD | KLK3 | MAPK12 | NDUFA7 | PC |
| GOT2 | HPGDS | KLRC3 | MAPK13 | NECTIN1 | PCK2 |
| GPATCH3 | HPRT1 | KLRC4 | MARCKSL1 | NECTIN2 | PCLAF |
| GPC4 | HS3ST2 | KRAS | MEGF9 | PVRL3 | PDE4A |
| GPNMB | HSD17B11 | KRBA1 | MELK | NEIL1 | PDGFA |
| GPR146 | HSPG2 | KYNU | MET | NF1 | PDGFB |
| GPR15 | ICAM5 | LAIR1 | MFNG | NFAM1 | PDGFRA |
| GPR160 | ID1 | LAMA1 | MGMT | NFIL3 | PDK1 |
| GPR183 | ID2 | LAMB2 | MKI67 | NFKBIE | PDZK1IP1 |
| GPR65 | ID3 | LAMB3 | MLANA | NFKBIZ | PELI1 |
| GPSM3 | ID4 | LAMC2 | MLH1 | NFYC | PF4 |
| GRN | IDO2 | LAPTM5 | MMP1 | NGF | PFDN6 |
| GSN | IER3 | LAT | MMP12 | NGFR | PFKFB3 |
| GTF2H2 | IFI6 | LAT2 | MMP13 | NID2 | PFKM |
| GUSB | IFI44 | LDHA | MMP19 | NKG7 | PGGT1B |
| H2AFX | IFI44L | LDHB | MMP7 | NLRP12 | PGLYRP1 |
| HAVCR1 | IFIT3 | LDLR | MMP8 | NMB | PGPEP1 |
| HBEGF | IFNGR2 | LGALS9 | MMP9 | NOTCH2 | PHEX |
| HDAC11 | IGF1 | LGMN | MMRN2 | NOX1 | PHLDA2 |
| HDAC3 | IGF2 | LILRA3 | MNDA | NOX3 | PIAS4 |
| HDAC4 | IHH | LILRB4 | MOK | NOX4 | PIK3CA |
| HDAC5 | IKZF1 | LIPA | MPEG1 | NR1H3 | PIK3R1 |
| HDAC6 | IL27RA | LOX1 | MPO | NR2F6 | PIK3R2 |
| HDC | IL33 | LOXL2 | MRC2 | NR4A2 | PIK3R5 |
| HELLS | IL4I1 | LPL | MRE11 | NRAS | PIM2 |
| HERC5 | INHBA | LRP5 | MRPL19 | NRDE2 | PKM |
| HERC6 | INSIG1 | LRP6 | MRPS5 | NRIP3 | PLA1A |
| HES1 | INSR | LRRC32 | MS4A4A | OAS1 | PLA2G2A |
| HEY1 | IRF9 | LST1 | MS4A6A | OAS2 | PLA2G7 |
| PLD4 | RAD50 | SELP | SPHK1 | TOP2A | WNT11 |
| PLOD2 | RAD51 | SEMA4A | SPIB | TPI1 | WNT2 |
| PLSCR1 | RAD51C | SEMA4B | SPRY2 | TPM1 | WNT2B |
| PLVAP | RAF1 | SEMA5A | SPRY4 | TPSAB1/B2 | WNT3A |
| PMS2 | RAPGEF4 | SEMA6D | SPTBN1 | TRAF1 | WNT4 |
| PNOC | RASAL1 | SERPINA1 | SQSTM1 | TRAFD1 | WNT5A |
| POLD1 | RASGRF1 | SERPINB5 | SREBF1 | TRANK1 | WNT5B |
| POLR2A | RASGRF2 | SERPINB9 | SRP54 | TRAT1 | WNT7B |
| PPARGC1B | RB1 | SERPINE1 | STAB1 | TREX1 | XBP1 |
| PRG3 | RBL2 | SERPINE3 | STAT5A | TRIM21 | XCL1/2 |
| PRKAA2 | RELN | SERPINH1 | STC1 | TSLP | YAP1 |
| PRKACB | REN | SF3A1 | STEAP4 | TSPAN8 | YES1 |
| PRKCA | RETNLB | SF3A3 | STK11IP | TTC30A | ZC3H12A |
| PRKCI | RGS1 | SFRP1 | STK17A | TUBA1A | ZC3H14 |
| PRKX | RGS6 | SFRP4 | TAF3 | TUBA4A | ZEB1 |
| PRLR | RHOC | SFXN1 | TAGLN | TWF1 | ZEB2 |
| PROK2 | RHOG | SGK1 | TAPBPL | TWIST1 | ZFP92 |
| PROM1 | RHOJ | SGPP1 | TBC1D10B | TWIST2 | ZNF148 |
| PROS1 | RICTOR | SHC2 | TBP | TWISTNB | ZNRF2 |
| PRPF38A | RIPK1 | SIGLEC5 | TBXAS1 | TXN | |
| PRR5 | RIPK3 | SIGLEC8 | TCF3 | TXNDC16 | |
| PRRX1 | RNASE2 | SIRPA | TCL1A | TYMP | |
| PSMB5 | RNASE3 | SIRPB2 | TDO2 | TYMS | |
| PSMC4 | RND3 | SKI | TEP1 | TYROBP | |
| PSME2 | RNLS | SKIL | TFCP2 | UBA7 | |
| PTAFR | ROBO4 | SLC16A1 | TGFA | UBB | |
| PTCD2 | ROCK1 | SLC16A3 | TGFB3 | UBE2C | |
| PTCRA | ROR2 | SLC16A6 | TGFBR1 | UBE2T | |
| PTEN | ROS1 | SLC1A5 | TGFBR2 | USP18 | |
| PTGDR | RPL23 | SLC2A1 | TGFBR3 | VAMP2 | |
| PTGDS | RPL7A | SLC7A5 | TGM2 | VASP | |
| PTGER2 | RPS6KB1 | SLCO2A1 | TIE1 | VAV1 | |
| PTGER4 | RPTOR | SMAD1 | TIMD4 | VAV2 | |
| PTGS1 | RRM2 | SMAD5 | TIMP3 | VCAN | |
| PTPN11 | RSAD2 | SMAD7 | TM4SF19 | VEGFB | |
| PTPN14 | RTP4 | SMAP1 | TM7SF3 | VNN3 | |
| PTPRB | RUNX2 | SMARCD3 | TMEM140 | VHL | |
| CD45RB | S100A10 | SNAI1 | TMEM173 | VRK2 | |
| CD45RA | S100A4 | SNCA | TMUB2 | VSIR | |
| CD45RO | S100A9 | SOCS2 | TNC | VTCN1 | |
| PTPRK | S1PR1 | SOCS3 | TNFAIP6 | VWA5A | |
| PTX3 | SAMD9 | SOX10 | TNFAIP8 | VWF | |
| PUM1 | SAMSN1 | SOX11 | TNFRSF10D | WAS | |
| PVRIG | SAP130 | SOX2 | TNFRSF25 | WDR76 | |
| RAB20 | SCIN | SP4 | TNFSF9 | WFDC21P | |
| RAB3D | SDHA | SPATS2L | TNKS | WNT10A | |
Supplementary Figure 2. Nanostring gene panel
