# Supplementary material for: Characterization of the Immunogenomic Landscape of Ovarian Cancer Uncovers a Distinct Subset of Endometroid Tumors Associated with High CST2 Expression and a Favorable Prognosis
Source: Cancer Res Commun. 2026 Jan 28;6(1):224–34. doi: 10.1158/2767-9764.CRC-25-0150 (PMC12848861; doi:10.1158/2767-9764.CRC-25-0150)
Supplement: Supplementary Figure 2 — Overall survival (a) and progression-free survival (b) curves for patients stratified by COLL1A1 expression. [file crc-25-0150_supplementary_figure_2_suppsf2.pptx]

## Slide 1
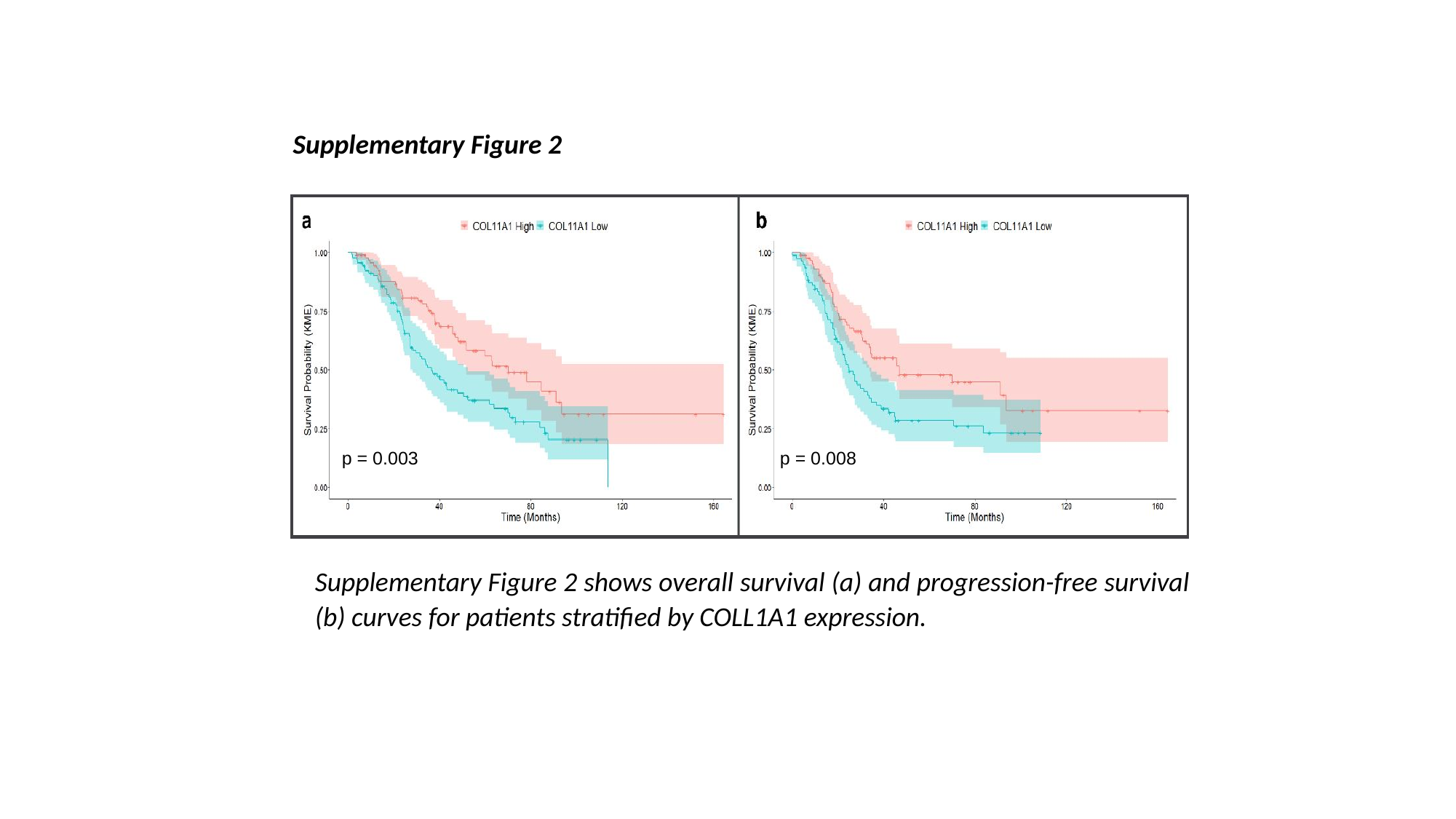

Supplementary Figure 2
p = 0.003
p = 0.008
Supplementary Figure 2 shows overall survival (a) and progression-free survival (b) curves for patients stratified by COLL1A1 expression.
