# Supplementary material for: Characterization of the Immunogenomic Landscape of Ovarian Cancer Uncovers a Distinct Subset of Endometroid Tumors Associated with High CST2 Expression and a Favorable Prognosis
Source: Cancer Res Commun. 2026 Jan 28;6(1):224–34. doi: 10.1158/2767-9764.CRC-25-0150 (PMC12848861; doi:10.1158/2767-9764.CRC-25-0150)
Supplement: Supplementary Figure 3 — Plots of T Cell Inhibitor versus Immune Cell density across different regions of the tumour. [file crc-25-0150_supplementary_figure_3_suppsf3.pptx]

## Slide 1
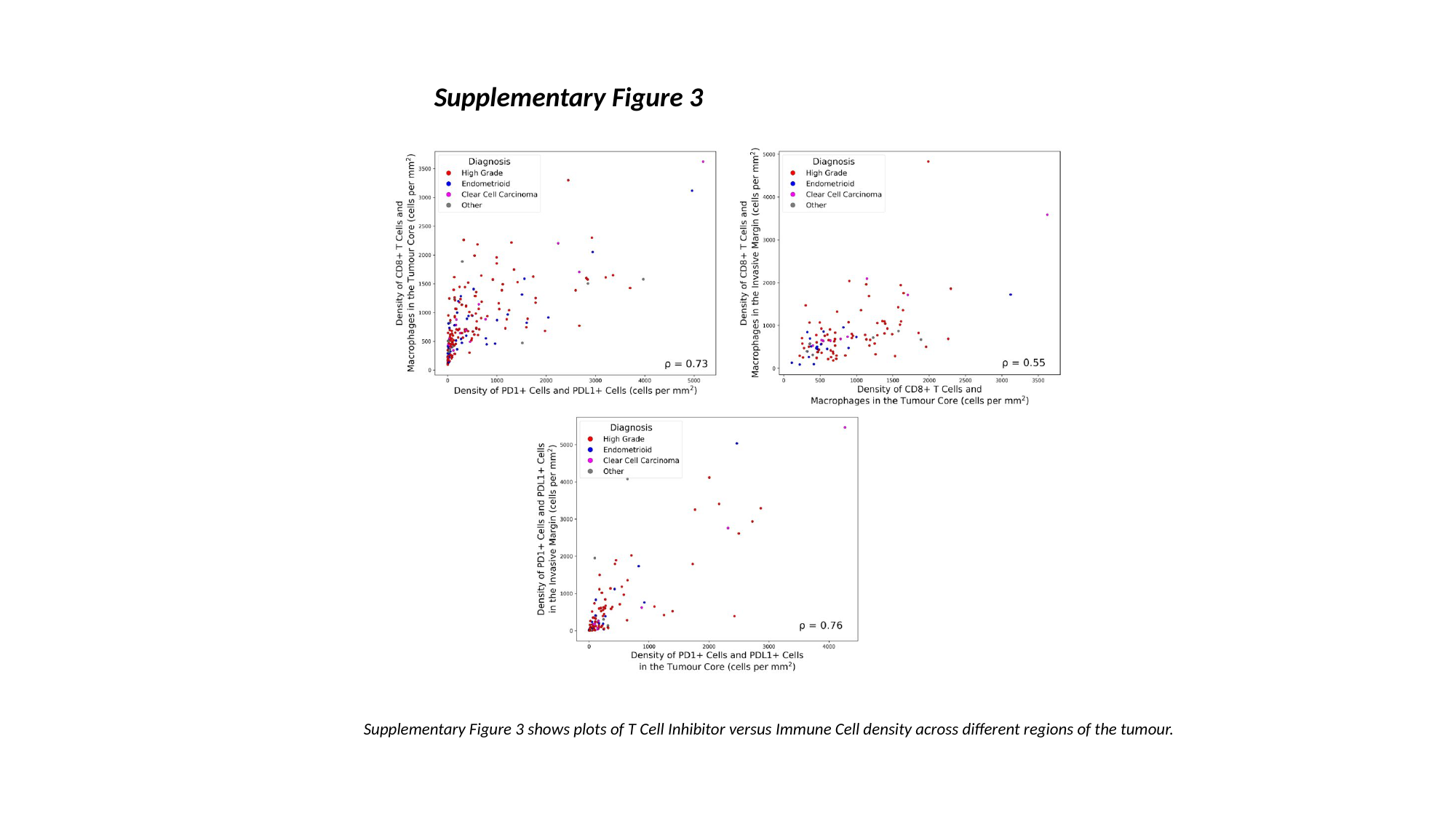

Supplementary Figure 3
Supplementary Figure 3 shows plots of T Cell Inhibitor versus Immune Cell density across different regions of the tumour.
